# Lung Tissue Microbiome Is Associated With Clinical Outcomes of Idiopathic Pulmonary Fibrosis

**DOI:** 10.3389/fmed.2021.744523

**Published:** 2021-10-18

**Authors:** Hee-Young Yoon, Su-Jin Moon, Jin Woo Song

**Affiliations:** Department of Pulmonary and Critical Care Medicine, Asan Medical Center, University of Ulsan College of Medicine, Seoul, South Korea

**Keywords:** idiopathic pulmonary fibrosis, prognosis, respiratory function tests, microbiota, diagnosis

## Abstract

**Background:** Several studies using bronchoalveolar lavage fluid (BALF) reported that lung microbial communities were associated with the development and clinical outcome of idiopathic pulmonary fibrosis (IPF). However, the microbial communities in IPF lung tissues are not well known. This study is aimed to investigate bacterial microbial communities in lung tissues and determine their impact on the clinical outcomes of patients with IPF.

**Methods:** Genomic DNA extracted from lung tissues of patients with IPF (*n* = 20; 10 non-survivors) and age- and sex-matched controls (*n* = 20) was amplified using fusion primers targeting the V3 and V4 regions of the 16S RNA genes with indexing barcodes.

**Results:** Mean age of IPF subjects was 63.3 yr, and 65% were male. Alpha diversity indices did not significantly differ between IPF patients and controls, or between IPF non-survivors and survivors. The relative abundance of *Lactobacillus, Paracoccus*, and *Akkermansia* was increased, whereas that of *Caulobacter, Azonexus*, and *Undibacterium* decreased in patients with IPF compared with that in the controls. A decreased relative abundance of *Pelomonas* (odds ratio [OR], 0.352, *p* = 0.027) and *Azonexus* (OR, 0.013, *p* = 0.046) was associated with a diagnosis of IPF in the multivariable logistic analysis adjusted by age and gender. Multivariable Cox analysis adjusted for age and forced vital capacity (FVC) revealed that higher relative abundance of *Streptococcus* (hazard ratio [HR], 1.993, *p* = 0.044), *Sphingomonas* (HR, 57.590, *p* = 0.024), and *Clostridium* (HR, 37.189, *p* = 0.038) was independently associated with IPF mortality. The relative abundance of *Curvibacter* (*r* = 0.590) and *Thioprofundum* (*r* = 0.373) was correlated positively, whereas that of *Anoxybacillus* (*r* = −0.509) and *Enterococcus* (*r* = −0.593) was correlated inversely with FVC. In addition, the relative abundance of the *Aquabacterium* (*r* = 0.616) and *Peptoniphilus* (*r* = 0.606) genera was positively correlated, whereas that of the *Fusobacterium* (*r* = −0.464) and *Phycicoccus* (*r* = −0.495) genera was inversely correlated with distance during the 6-min walking test.

**Conclusions:** The composition of the microbiome in lung tissues differed between patients with IPF and controls and was associated with the diagnosis, mortality, and disease severity of IPF.

## Introduction

Idiopathic pulmonary fibrosis (IPF) is a chronic progressive fibrosing interstitial lung disease of unknown etiology (1). It is characterized by worsening dyspnea, impaired lung function, decreased quality of life, and a poor prognosis (1). The pathogenesis of IPF involves both genetic (2, 3) and environmental factors (4, 5). Repeated epithelial injuries caused by multiple environmental factors, such as smoking, micro-aspiration, organic and inorganic dust, and viral infection (4, 5), can lead to the abnormal wound healing process, such as epithelial-mesenchymal transition (6) in genetically susceptible individuals who have a mutation in airway defense (*MUC5B*), telomerase function (*TERT*), or immune responses (*TOLLIP, TLR3*, and *IL1RN*) (2, 3, 7). Much evidence supports an association between the etiology of several viruses (8–11), and the development or acute exacerbation (AE) of IPF (12, 13). The fact that combined therapy with steroid, azathioprine, and N-acetylcysteine increases the mortality and hospitalization rates of patients with IPF (14) also suggests that infectious organisms are involved in IPF progression.

Along with the development of culture-independent molecular-sequencing techniques, such as 16s ribosomal RNA (16s rRNA) gene sequencing (15), several studies of bronchoalveolar lavage fluid (BALF) have suggested that lung microbial communities are associated with the clinical course of IPF (16–21). The findings of the Correlating Outcomes With Biochemical Markers to Estimate Time-progression in IPF (COMET) study revealed that an increased bacterial burden in BALF from patients with IPF (*n* = 65), compared with controls (*n* = 44), is associated with a 10% decline in forced vital capacity (FVC) at 6 months and mortality (16). On the contrary, a study of explanted lung tissues from patients with IPF (*n* = 40) showed very low bacterial abundance in IPF lung tissues that was similar to that of negative controls (22). These contradictory findings could be attributed to different types of samples or sample collection times. Therefore, the composition and impact of the lung tissue microbiome at diagnosis on clinical outcomes in patients with IPF are not well defined. Our study aimed to identify the diversity and composition of the bacterial microbial communities in lung tissues at the time of diagnosis and determine their association with clinical outcomes, such as survival, disease severity, and progression in patients with IPF.

## Materials and Methods

### Study Population

All participating patients with IPF were diagnosed between January 2011 and December 2013 at Asan Medical Center, Seoul, Republic of Korea and met the diagnostic criteria of the American Thoracic Society (ATS)/European Respiratory Society/Japanese Respiratory Society/Latin American Thoracic Association statement (1). Samples of lung tissues from patients with IPF (*n* = 20; 10 non-survivors [cause of death: AE = 1, disease progression = 2, unknown = 7]) were aseptically obtained at the time of surgical biopsy for diagnosis, and those from age- and gender-matched controls (lung cancer patients; *n* = 20) with no histological evidence of disease collected aseptically at the time of surgery were obtained from the Bio-Resource Center of Asan Medical Center. None of the patients with IPF or the controls had been treated with antibiotics, steroids, anti-fibrotic agents, or probiotics within 1 month before undergoing surgery. Lung tissues were procured under protocol #2016-1366. This study was conducted in accordance with the Declaration of Helsinki (2013) and was approved by the Institutional Review Board of Asan Medical Center (2018–1096). Written informed consent was obtained from all study participants.

Clinical and survival data of all patients were retrospectively collected from medical records, telephone interviews, and/or the National Health Insurance of Korea. Spirometry, total lung capacity (TLC) determined by plethysmography and diffusing capacity for carbon monoxide (DLco) measured according to published recommendations are expressed as ratios (%) of normal predicted values (23–25). The patients with IPF underwent 6-min walk tests (6MWT) according to the ATS guidelines (26). Baseline clinical data at the time of IPF diagnosis were collected within one month of sample acquisition.

### Bacterial 16S rRNA Gene Sequencing

Tissue samples were frozen in liquid nitrogen immediately after collection and stored at −80°C. Genomic DNA was extracted from lung tissues using Mo Bio PowerSoil® DNA Isolation Kits (Mo Bio Laboratories, Carlsbad, CA, USA) according to the instructions of the manufacturer. The variable V3 and V4 regions of the 16S rRNA genes were amplified using the following specific forward and reverse primers with overhang adapters: 5′-TCGTCGGCAGCGTCAGATGTGTATAAGAGACAGCCTACG GGNGGCWGCAG-3′ and 5′-GTCTCGTGGGCTCGGAGATGTGTATAAGAGACAGGACTACHVG GGTATCTAATCC-3′, respectively (27). The PCR proceeded using a 5 ng/μl DNA template, 2 × KAPA HiFi HotStart Ready Mix (KAPA Biosystems, Wilmington, MA, USA), and two amplicon PCR forward and reverse primers. The PCR protocol comprised initial incubation at 95°C for 3 min, followed by 25 cycles of 95°C for 30 s, 55°C for 30 s, and 72°C for 30 s, then 72°C for 5 min, and retention at 4°C. After PCR clean-up and index PCR, 300 bp paired-end sequences were pooled on the Illumina MiSeq platform (Illumina Inc., San Diego, CA, USA) as described by the manufacturer for all sample sequencing (28, 29). Distilled water provided in the PCR kit was used as a negative control, and no amplification was identified during the processes.

### Reconstruction and Compositional Analysis

Fast Length Adjustment of Short (FLASH) reads, http://ccb.jhu.edu/software/FLASH/), were used for 16S rRNA gene by merging pairs of reads when the original DNA fragments were shortened than two times the length of the reads (30). Pre-processing and clustering were performed using the CD-HIT-operational taxonomic units (OTU; http://weizhongli-lab.org/cd-hit-otu/). Short reads (56,825) were filtered out and extra-long tails were trimmed. After filtering, the remaining reads were clustered at 100% identity. Chimeric reads (254,891) were filtered, and secondary clusters were recruited into primary clusters. After excluding reads with all other noise (5,292,165), the remaining reads (3,484,551) were clustered algorithm into OTUs at a cutoff of 97% (31, 32). Feature tables, such as abundance and representational sequence files, were created using UCLAST in Quantitative Insights Into Microbial Ecology (QIIME1; https://qiime.org) software (33). Taxonomy was assigned based on information about organisms with the closest similarity to the representative sequence of each OTU in the Basic Local Alignment Search Tool (BLAST), version 2.4.0, the NCBI 16S microbial reference database. Taxonomy was not assigned when the query coverage of the best match in the database was <85%, and the identity of the matched area was <85%.

### Statistical Analysis

Continuous data were analyzed using Mann–Whitney U tests, and categorical data were analyzed using Fisher exact tests. The decline rate of lung function and exercise capacity for one year was estimated by linear regression analysis. Correlations between the relative abundance of the microbiome and clinical parameters were assessed using Spearman's correlation coefficients (*r*). The risk of microbial relative abundance for a diagnosis of IPF was expressed as odds ratio (OR) with 95% CI using binary logistic regression. In addition, the risk of microbial relative abundance for IPF mortality was presented as hazard ratio (HR) with 95% CI using Cox proportional hazards regression analyses. Alpha diversity indices that estimate the number of unique OTU in each sample are represented using four indices; Observed estimated the actual number of different taxa evident in a sample, Chao 1 non-parametrically estimated the richness of the species (34), Shannon estimated richness and evenness of species present in a sample considering the distribution of strains belonging to each species (35), and Inverse Simpson measured the probability that two randomly selected objects in a sample belong to the same species (36). Principal coordinates analysis (PCoA), based on weighted UniFrac methods to obtain phylogenetic and quantitative indices for assessing abundance differences among groups (IPF vs. controls, survivors vs. non-survivors), was conducted for all samples using QIIME1 (37). The exploratory and differential microbial compositions were analyzed using QIIME1. All data were statistically analyzed using SPSS version 24.0 (IBM Corp., Armonk, NY, USA), and values with *p* < 0.05 (two-tailed) were considered statistically significant.

## Results

### Microbial Diversity and Composition

Among 20 patients with IPF, the mean age was 63.3 yr, and 65.0% were male (Table 1). Lung function (FVC, DLco, and TLC) was worse in the patients with IPF than in the controls, whereas demographics, lung function, and exercise capacity during the 6MWT did not significantly differ between IPF non-survivors and survivors.

**Table 1:** Comparison of baseline characteristics between IPF and control groups.

**Variables**	**IPF**			**Control**
	**Total**	**Survivors**	**Non-survivors**	
Number	20	10	10	20
Age, years	63.3 ± 6.2	62.4 ± 6.4	64.2 ± 6.1	67.3 ± 7.4
Male	13 (65.0)	6 (60.0)	7 (70.0)	17 (85.0)
Ever-Smoker	13 (65.0)	7 (70.0)	6 (60.0)	15 (75.0)
PFT, % predicted				
FVC	64.2 ± 14.7	69.2 ± 15.1	60.2 ± 14.0	91.3 ± 16.0^*^
DLco	54.8 ± 14.8	58.3 ± 17.5	51.3 ± 11.3	97.4 ± 20.6^*^
TLC	65.9 ± 11.3	68.7 ± 10.2	63.0 ± 12.2	108.4 ± 17.7^*^
6MWT				
Distance, m	433.6 ± 63.5	452.8 ± 48.9	434.3 ± 77.1	NA
Resting SpO_2_, %	96.3 ± 1.6	96.4 ± 1.6	96.2 ± 1.7	NA
Lowest SpO_2_, %	90.6 ± 5.8	91.5 ± 5.1	89.6 ± 6.5	NA

*Data are presented as means ± SD or number (%), unless otherwise indicated. 6MWT, 6-min walk test; DLco, diffusing capacity of the lungs for carbon monoxide; FVC, forced vital capacity; IPF, idiopathic pulmonary fibrosis; NA, not available; PFT, pulmonary function test; SpO_2_, peripheral oxygen saturation; TLC, total lung capacity; 6MWD, 6-min walk test distance*.

* *P < 0.05 (IPF vs. control)*.

**Figure 1:**
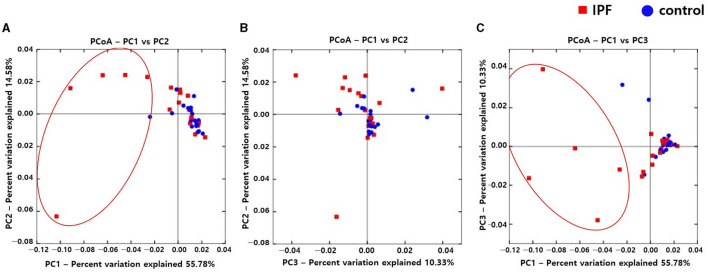
Comparison of principal coordinates analysis using weighted UniFrac method between patients with IPF and controls. Two-dimensional PCoA plots display inter-sample distances by three principal coordinates as PC1 and PC2 **(A)**, PC2 and PC3 **(B)**, and PC1 and PC3 **(C)**. Each dot represents one sample, plotted by a principal component on the *X*-axis and another principal component on the *Y*-axis, and colored by the group. The ratio (%) on each axis presents the contribution of values to discrepancies among samples. IPF, idiopathic pulmonary fibrosis; PCoA, principal coordinates analysis.

Alpha diversity indexes, such as Observed, Chao 1, Shannon, and Inverse Simpson, did not differ between the IPF and control groups (Supplementary Figure A1). However, the PCoA plot revealed dissimilarity in the weighted UniFrac distance between the IPF and controls (Figures 1A–C), especially between five of the patients with IPF (non-survivors, *n* = 3; Figures 1A,C red circles) and controls, indicating more heterogeneity in the microbial distribution.

**Figure 2:**
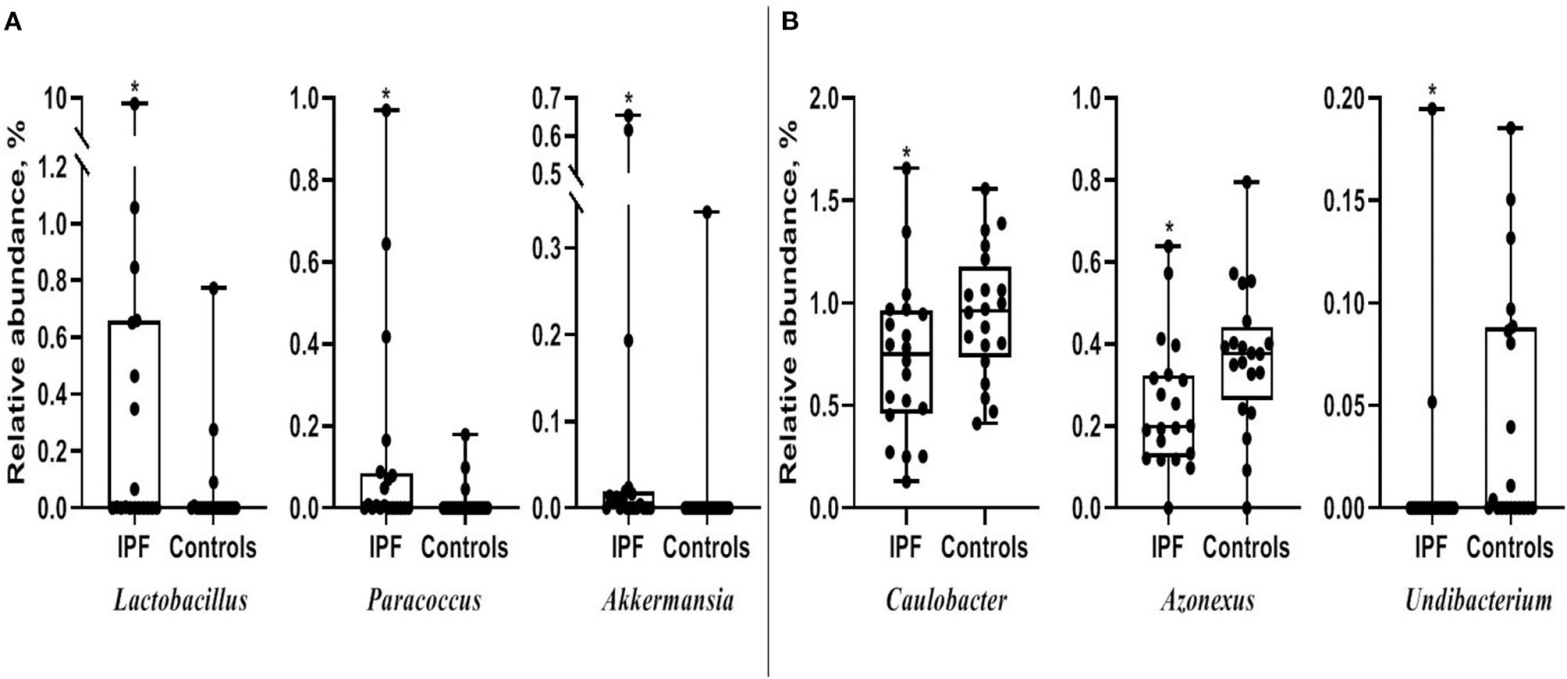
Comparison of relative abundance between patients with IPF and controls. **(A)** Increased and **(B)** decreased in patients with IPF. The box plot shows the minimum, first quartile, median, third quartile, and maximum of relative abundance. *p < 0.05. IPF, idiopathic pulmonary fibrosis.

Among the 10 most frequent taxa, the genus *Ralstonia* was the most prevalent in the IPF and control groups, followed by *Nocardia* and *Pelomonas* (Supplementary Figure A2). On the contrary, *Lactobacillus, Enterobacter, Tetragenococcus*, and *Neisseria* were frequently identified in IPF, whereas *Haemophilus, Caulobacter, Bradyrhizobium*, and *Thermomonas* were prevalent in the controls. The relative abundance of *Lactobacillus* (0.91 [IPF] vs. 0.06% [control], *p* = 0.009)*, Paracoccus*, (0.13 vs. 0.02%, *p* = 0.013), and *Akkermansia* (0.08 vs. 0.02%, *p* = 0.001) was higher, whereas that of *Caulobacter* (0.73 vs. 0.95%, *p* = 0.040)*, Azonexus* (0.25 vs. 0.37%, *p* = 0.021), and *Undibacterium* (0.01 vs. 0.04%, *p* = 0.011) was lower in patients with IPF than in the controls (Figure 2). Logistic analysis adjusted by age and gender independently associated a diagnosis of IPF with lower relative abundance of the genera, *Pelomonas* (OR, 0.352; 95% CI, 0.139–0.891; *p* = 0.027), and *Azonexus* (OR, 0.013; 95% CI, 0.000–0.926; *p* = 0.046; Table 2).

**Table 2:** Predictive factors for IPF diagnosis assessed by multivariable logistic regression.

**Genus**	OR (95% CI)^*^	***P*-value**
*Pelomonas*	0.352 (0.139–0.891)	0.027
*Dyella*	0.277 (0.067–1.139)	0.075
*Caulobacter*	0.154 (0.021–1.105)	0.063
*Lactobacillus*	12.881 (0.666–249.192)	0.091
*Bradyrhizobium*	0.051 (0.002–1.102)	0.058
*Azonexus*	0.013 (0.000–0.926)	0.046

*OR, odds ratio per 1% increase in relative abundance; IPF, idiopathic pulmonary fibrosis*.

* *Adjusted by age and gender*.

### Microbial Communities: IPF Non-survivors vs. Survivors

**Figure 3:**
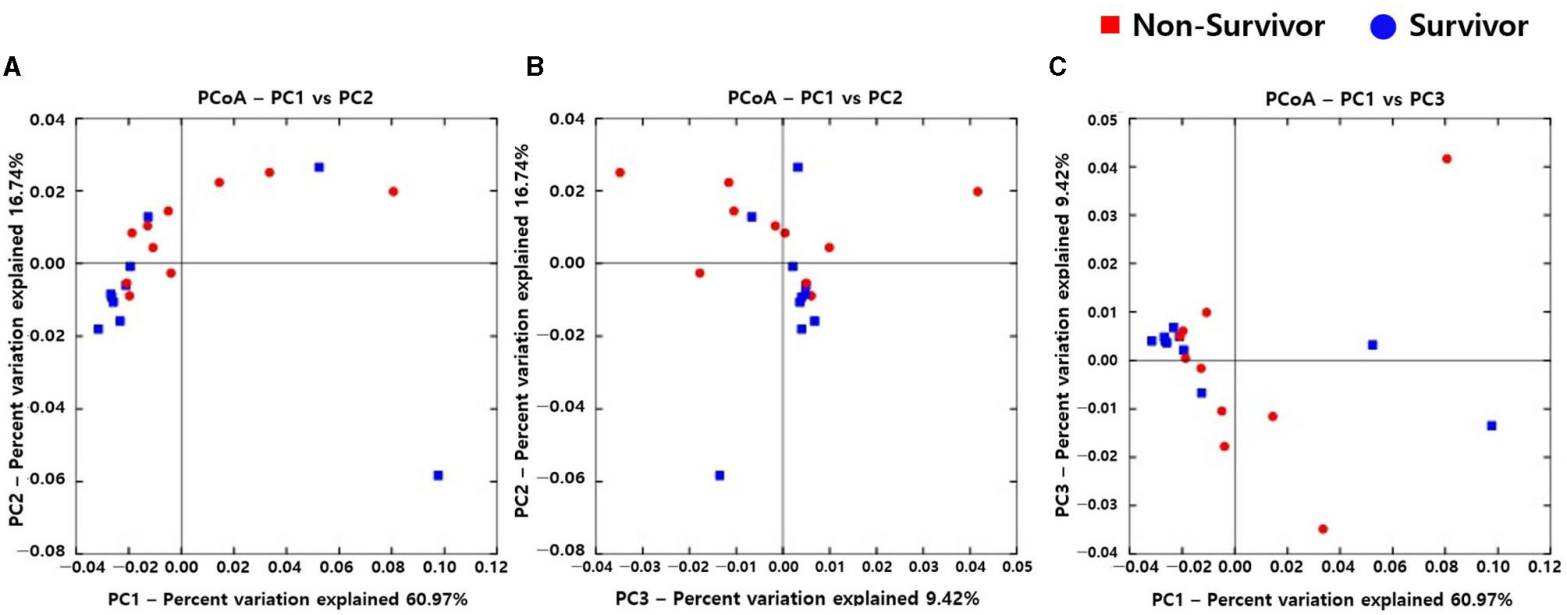
Comparison of principal coordinates analysis outcomes using weighted UniFrac between non-survivors and survivors of IPF. Two-dimensional PCoA plots display inter-sample distances by three principal coordinates as PC1 and PC2 **(A)**, PC2 and PC3 **(B)**, and PC1 and PC3 **(C)**. Each dot represents one sample, plotted by a principal component on the *X*-axis and another principal component on the *Y*-axis, and colored by the group. The ratio (%) on each axis presents the contribution of values to discrepancies among samples. IPF, idiopathic pulmonary fibrosis; PCoA, principal coordinates analysis.

**Figure 4:**
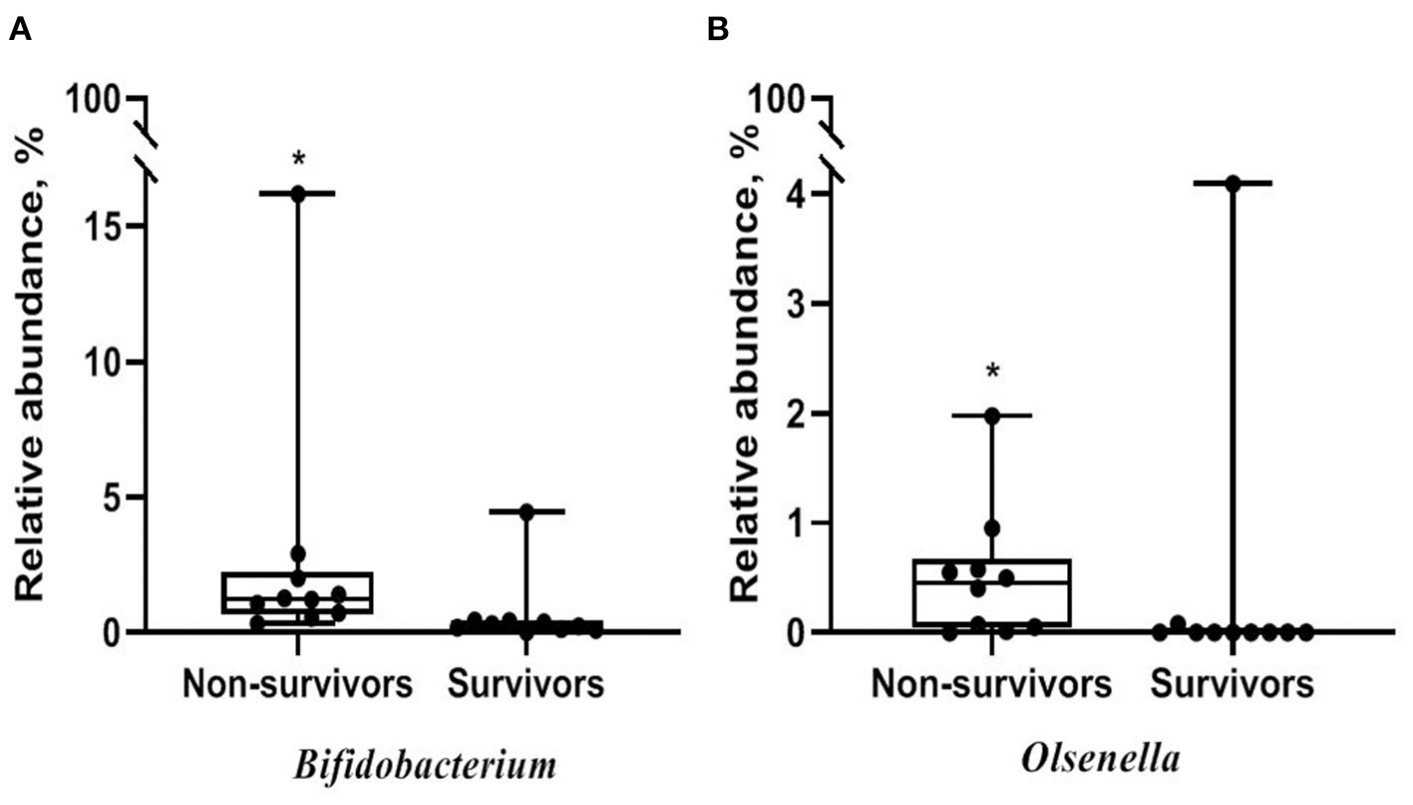
Comparison of relative abundance of microbes between non-survivors and survivors of IPF. **(A)** Genus *Bifidobacterium* **(B)** genus *Olsenella*. The box plot represents minimum, first quartile, median, third quartile, and maximum relative abundance. IPF, idiopathic pulmonary fibrosis. *p < 0.05.

Alpha diversity did not significantly differ between non-survivors and survivors of IPF (Supplementary Figure A3). However, the PCoA plot showed that the distribution of microbes differed between non-survivors and survivors (Figure 3). Among the 10 most frequent taxa, *Ralstonia* and *Nocardia* were the most common in both groups (Supplementary Figure A4). The genus *Streptococcus* was more abundant in non-survivors compared with survivors. In addition, the genera *Neisseria, Haemophilus, Rothia*, and *Rubrobacter* were frequently detected in non-survivors, while the genera *Tetragenococcus, Enterobacter, Lactobacillus*, and *Caulobacter* were prevalent in survivors. The relative abundance of genera *Bifidobacterium* (2.77 [non-survivors] vs. 0.68% [survivors], *p* = 0.003) and *Olsenella* (0.51 vs. 0.41%, *p* = 0.013) was significantly higher in non-survivors than in survivors (Figures 4A,B).

### Impact on Survival

The median follow-up period for patients with IPF was 3.0 yr (interquartile range: 1.5–5.4 yr), and the median survival period was 3.1 yr. Unadjusted Cox analysis significantly associated the relative abundance of the *Streptococcus, Sphingomonas, Veillonella*, and *Clostridium* genera with IPF mortality. *Neisseria* and *Granulicatella* were also marginally associated with IPF mortality (Table 3). A multivariable model adjusted for age, and FVC selected a higher relative abundance of the *Streptococcus* (HR, 1.993; 95% CI, 1.019–3.901; *p* = 0.044), *Sphingomonas* (HR, 57.590; 95% CI, 1.714–1934.881; *p* = 0.024), and *Clostridium* (HR, 37.189; 95% CI, 1.228–1126.474; *p* = 0.038) genera as independent predictors of IPF mortality.

**Table 3:** Risk factors for mortality in patients with IPF assessed using multivariable Cox proportional hazards models.

**Genus**	**Unadjusted**		Multivariable^*^	
	**HR (95% CI)**	***P*-value**	**HR (95% CI)**	***P*-value**
*Streptococcus*	1.389 (1.047–1.842)	0.023	1.993 (1.019–3.901)	0.044
*Neisseria*	1.500 (0.937–2.400)	0.091	1.500 (0.937–2.400)	0.091
*Sphingomonas*	57.590 (1.714–1934.881)	0.024	57.590 (1.714–1934.881)	0.024
*Veillonella*	3.164 (1.026–9.752)	0.045	5.855 (0.821–41.769)	0.078
*Granulicatella*	14.029 (0.668–294.603)	0.089	14.029 (0.668–294.603)	0.089
*Clostridium*	37.189 (1.228–1126.474)	0.038	37.189 (1.228–1126.474)	0.038

*CI, confidence interval; HR, hazard ratio*.

* *Adjusted by age and FVC. FVC, forced vital capacity; IPF, idiopathic pulmonary fibrosis*.

### Association with Disease Severity

**Figure 5:**
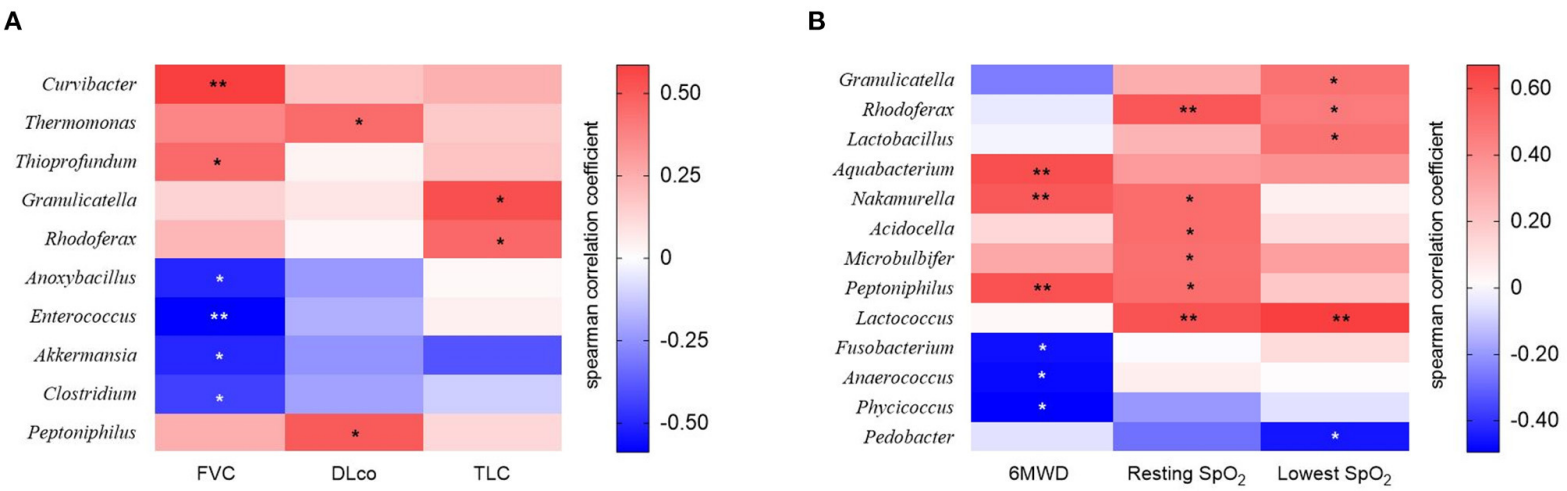
The heatmap showing correlations between relative abundance of bacterial taxa and **(A)** lung function or **(B)** exercise capacity in patients with IPF. Spearman's correlations between the relative abundances of the bacterial genera and lung function and exercise capacity at diagnosis were calculated. Blue: negative correlations; red: positive correlations. *p < 0.05, ** *p* < 0.01. DLco, diffusing capacity of the lung for carbon monoxide; FVC, forced vital capacity; IPF, idiopathic pulmonary fibrosis; TLC, total lung capacity. 6MWD, 6-minute walk test distance; SpO_2_, oxygen saturation.

The relative abundance of *Curvibacter* and *Thioprofundum* was positively associated with FVC in patients with IPF, whereas *Anoxybacillus, Enterococcus, Akkermansia*, and *Clostridium* negatively correlated with FVC (Figure 5A and Supplementary Table A1). The relative abundance of *Thermomonas* and *Peptoniphilus* positively correlated with DLco, whereas that of *Granulicatella* and *Rhodoferax* was positively correlated with TLC.

The relative abundance of the *Aquabacterium, Nakamurella*, and *Peptoniphilus* genera was positively correlated, whereas that of the *Fusobacterium, Anaerococcus*, and *Phycicoccus* genera was negatively correlated with distance during the 6MWT (Figure 5B and Supplementary Table A2). The relative abundance of genus *Rhodoferax* and *Lactococcus* was positively correlated with resting and lowest oxygen saturation (SpO_2_) during the 6MWT.

### Association with Disease Progression

**Figure 6:**
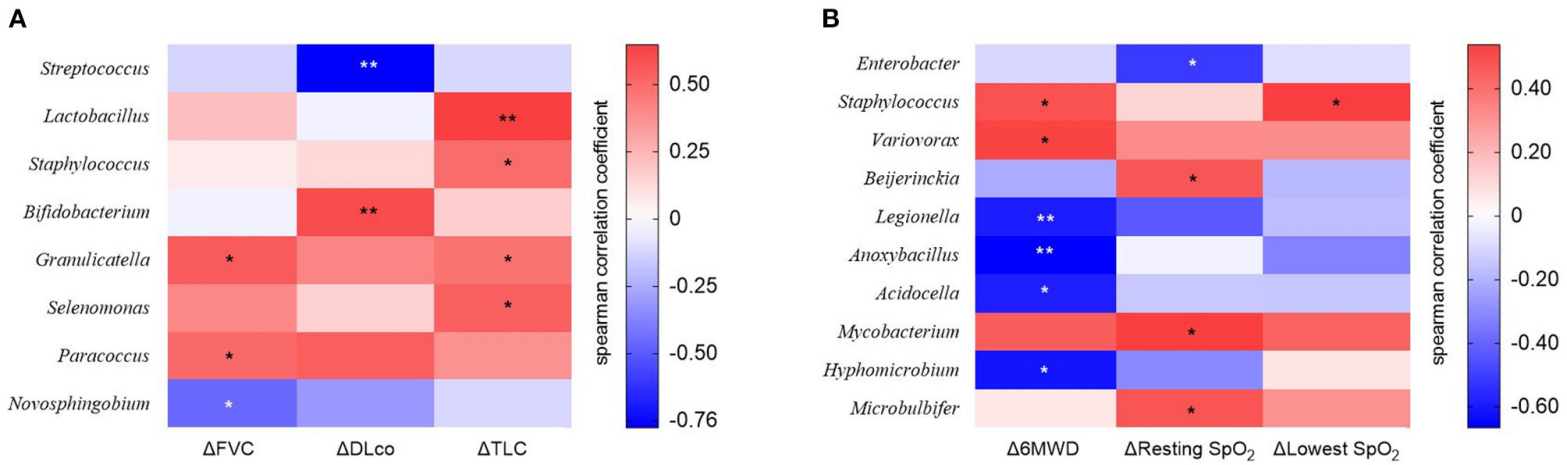
The heatmap showing correlations between relative abundance of bacterial taxa and **(A)** changes in lung function or **(B)** exercise capacity in patients with IPF. Spearman's correlations between the relative abundances of the bacterial genera and decline rate in lung function and exercise capacity for 1 year after diagnosis were calculated. Blue: negative correlations; red: positive correlations. *p < 0.05, ** *p* < 0.01. DLco, diffusing capacity of the lung for carbon monoxide; FVC, forced vital capacity; IPF, idiopathic pulmonary fibrosis; TLC, total lung capacity. 6MWD, 6-minute walk test distance; SpO_2_, oxygen saturation; Δ, decline rate for 1 year.

We estimated the decline rate in lung function and exercise capacity for one yr and compared them between survivors and non-survivors (Table 4). Non-survivors had a faster decline rate in DLco, and distance and the lowest SpO_2_ during 6MWT compared with survivors. The relative abundance of *Granulicatella* and *Paracoccus* genera was positively correlated, while that of the *Novosphingobium* genus was negatively correlated with the decline rate in FVC (Figure 6A and Supplementary Table A3). The relative abundance of *Bifidobacterium* was positively associated, whereas *Streptococcus was* negatively associated with the decline rate in DLco. The relative abundance of *Lactobacillus, Staphylococcus, Granulicatella*, and *Selenomonas* genera was positively correlated with the decline rate of TLC.

**Table 4:** Comparison of changes in lung function and exercise capacity between the survivors and non-survivors among patients with IPF.

	**Total**	**Survivors**	**Non-survivors**
FVC %predicted/year	−0.01 ± 0.04	0.00 ± 0.38	−0.02 ± 0.47
DLco %predicted/year	−0.03 ± 0.05	0.00 ± 0.03	−0.05 ± 0.05^*^
TLC %predicted/year	0.00 ± 0.04	0.00 ± 0.02	0.00 ± 0.06
6MWD, meter/year	−0.27 ± 0.47	−0.03 ± 0.23	−0.51 ± 0.53^*^
Resting SpO_2_, %/year	0.00 ± 0.02	0.00 ± 0.01	0.00 ± 0.02
Lowest SpO_2_, %/year	−0.01 ± 0.02	0.00 ± 0.02	−0.02 ± 0.02^*^

*Data are presented as means ± standard, unless otherwise indicated. 6MWT, 6-min walk test; DLco, diffusing capacity of the lungs for carbon monoxide; FVC, forced vital capacity; IPF, idiopathic pulmonary fibrosis; PFT, pulmonary function test; SpO_2_, peripheral oxygen saturation; TLC, total lung capacity; 6MWD, 6-min walk test distance*.

* *p < 0.05 (survivors vs. non-survivors)*.

The relative abundance of *Staphylococcus* and *Variovorax* was positively associated, while that of *Legionella, Anoxybacillus, Acidocella*, and *Hyphomicrobium* genera was negatively associated with the decline rate in distance during 6MWT (Figure 6B and Supplementary Table A4). The relative abundance of *Beijerinckia, Mycobacterium*, and *Microbulbifer* genera was positively correlated, whereas that of *Enterobacter genus was* negatively correlated with the decline rate in resting SpO_2_. The relative abundance of genus *Staphylococcus* was positively correlated with the lowest SpO_2_ during 6MWT.

## Discussion

The microbial communities in the lung tissues differed between patients with IPF and controls, and between IPF non-survivors and survivors. When adjusted for age and gender, a decreased relative abundance of genus *Pelomonas* and *Azonexus* was associated with a diagnosis of IPF. A higher relative abundance of the *Streptococcus, Sphingomonas*, and *Clostridium* genera was an independent prognostic factor in patients with IPF and several genera correlated with disease severity and progression.

We found no differences in the alpha diversity of lung tissue microbiomes between patients with IPF and controls, whereas other results of studies of BALF from patients with IPF yielded different results (16, 19). Molineux et al. found a significantly decreased alpha diversity index for the microbiome in BALF samples from 65 patients with IPF at the time of diagnosis (Shannon diversity index, 3.81 ± 0.08 vs. 4.11 ± 0.10; *p* = 0.005) compared with controls (*n* = 44) (16). The Shannon diversity index was also decreased in BALF from mice treated with bleomycin (*n* = 6), compared with control mice (*n* = 6, *p* < 0.05) (19). These contradictory findings could be attributed to differences in baseline demographics and treatment of the subjects in a previous study (16); there were differences in age (68 [IPF] vs. 58.2 years [controls], *p* < 0.0001) and inhaled steroid therapy (6.2 vs. 0.0%, *p* = not significant) between IPF and controls, and these might affect differences in alpha diversity in microbiome. Our findings were in line with those of Kitsios et al. who identified separate clusters on PCoA plots of Bray-Curtis dissimilarity distances among explanted lung tissues from patients with IPF (n = 40), cystic fibrosis (*n* = 5), and donors (*n* = 7) (22).

The relative abundance of *Lactobacillus* was increased in lung tissues from patients with IPF compared with controls. *Lactobacillus* generally resides in the gastrointestinal and reproductive tract, where it maintains a healthy microecology with lactic acid production (38, 39). However, given the well-known association between IPF and gastroesophageal reflux disease (GERD) (40), the high prevalence of GERD in IPF might contribute to the increase in the relative abundance of *Lactobacillus* in IPF. Moreover, Harata et al. found the increased expression of the mRNA for interleukin-1, tumor necrosis factor, and monocyte chemotactic protein-1 in the respiratory tracts of mice infected with influenza and treated with intranasal *Lactobacillus rhamnosus* than in infected and untreated mice (41). Since proinflammatory cytokines and chemokines are associated with the pathogenesis of IPF (42), the immunoregulatory effect of *Lactobacillus* might contribute to the pathophysiology of IPF. We found a higher relative abundance of *Bifidobacterium* in IPF non-survivors than survivors. *Bifidobacterium* can also produce lactic acid (43, 44), along with *Lactobacillus*. Levels of lactic acid and lactate dehydrogenase-5, which induce the differentiation of fibroblasts into myofibroblasts by activating transforming growth factor (TGF)-ß1, were elevated in lung tissues from patients with IPF (*n* = 6), compared with healthy persons (*n* = 6) (45). Therefore, bacteria that produce lactic acid might also contribute to the progression of IPF.

We independently associated a higher relative abundance of the *Streptococcus* genus with IPF mortality, which is in line with the previous reports (21, 46–48). The COMET-IPF study of 55 patients with IPF found that a higher relative abundance of *Streptococcus* OTU was an independent prognostic factor for disease progression (HR, 1.11; 95% CI, 1.04–1.18; *p* = 0.0009) according to a multivariable Cox proportional hazard analysis adjusted for age, gender, smoking status, and desaturation during the 6MWT (21). Infection with *Streptococcus pneumoniae* significantly increased hydroxyproline levels in lung tissues from mouse models of TGFß1-induced lung fibrosis compared with mock infection (46). In addition, fibrosis and collagen deposition were increased in lung tissues from mice treated with both bleomycin and *Streptococcus pneumoniae* serotype 3 compared with mice that were either treated with bleomycin or infected with *Streptococcus pneumoniae* (48). These results suggested that *Streptococcus* infection could induce IPF disease progression. Furthermore, lung vascular permeability and neutrophil and monocytes counts were increased in BALF from mice treated with pneumolysin (47), which is a pore-forming cytotoxin released by *Streptococcus pneumoniae* that causes alveolar epithelial injury (47).

In this study, the relative abundance of the *Anoxybacillus* genus in IPF lung tissues was correlated with IPF disease severity. The relative abundance of the *Firmicutes* phylum was inversely correlated with FVC in BALF samples from 34 patients with IPF (*r* = −0.5514, *p* = 0.0007) (19). Our results are consistent with these findings because *Anoxybacillus* belongs to the *Firmicutes* phylum and is negatively correlated with FVC. Another study also found an increased relative abundance of *Firmicutes* in BALF from mice treated with bleomycin (19), suggesting that an increased prevalence of the *Anoxybacillus* genus is associated with the pathogenesis of IPF.

This study had some limitations. Although we matched the baseline characteristics, such as age and sex between the IPF and control groups, other confounding factors might have affected the microbial communities. However, we tried not to include patients who had been treated with agents that might affect the microbiota. The number of samples analyzed was not large. Nevertheless, we identified significant differences in the distribution and clinical impact of the microbiomes of patients with IPF compared with controls. Because non-malignant and non-fibrotic lung tissues from lung cancer patients were used as controls, the microbial community in the control group might be affected by lung cancer. However, in studies of lung tissue microbiome of other diseases, it is common to use normal tissue of lung cancer tissue as normal control (49, 50). Even in lung tissue microbiome studies in lung cancer patients, non-cancer tissue from the lung cancer patient was used as a control (50). The cross-sectional design of this study prevented the identification of causal relationships between changes in microbial communities and IPF development. Additional long-term clinical studies should address this issue. Despite these limitations, the strength of our study is that we first revealed the microbial communities in lung tissues from patients when they were initially diagnosed with IPF and the impact of these communities on their survival.

In conclusion, our finding suggests that specific microbial communities in lung tissues from patients with IPF and associations between the relative abundance of some genera and clinical parameters, such as diagnosis, mortality, disease severity, and progression in such patients, imply microbial communities in the lungs play roles in the pathogenesis of IPF.

## Data Availability Statement

The datasets presented in this study can be found in online repositories. The names of the repository/repositories and accession number(s) can be found below: NCBI SRA BioProject, accession no: PRJNA761508.

## Ethics Statement

The studies involving human participants were reviewed and approved by Institutional Review Board of Asan Medical Center (2018-1096). Written informed consent for participation was not required for this study in accordance with the national legislation and the institutional requirements.

## Author Contributions

JS was the guarantor of the manuscript for designing and supervising the entire study. H-YY and JS took responsibility for the data analysis. S-JM contributed to sample collection and preparation. H-YY and JS drafted the initial manuscript. All the authors discussed the results and reviewed the manuscript.

## Funding

This study was supported by a grant from the Basic Science Research Program of the National Research Foundation of Korea (NRF), which is funded by the Ministry of Science and Technology (NRF-2019R1A2C2008541), South Korea. The sponsor had no role in the design of the study, the collection and analysis of the data, or the preparation of the manuscript.

## Conflict of Interest

The authors declare that the research was conducted in the absence of any commercial or financial relationships that could be construed as a potential conflict of interest.

## Publisher's Note

All claims expressed in this article are solely those of the authors and do not necessarily represent those of their affiliated organizations, or those of the publisher, the editors and the reviewers. Any product that may be evaluated in this article, or claim that may be made by its manufacturer, is not guaranteed or endorsed by the publisher.

## References

[1] Raghu G Collard HR Egan JJ Martinez FJ Behr J Brown KK. An official ATS/ERS/JRS/ALAT statement: idiopathic pulmonary fibrosis: evidence-based guidelines for diagnosis and management. Am J Respir Crit Care Med. (2011) 183:788–824. 10.1164/rccm.2009-040GL 21471066 PMC5450933

[2] Baratella E Ruaro B Giudici F Wade B Santagiuliana M Salton F. Evaluation of correlations between genetic variants and high-resolution computed tomography patterns in idiopathic pulmonary fibrosis. Diagnostics. (2021) 11:762. 10.3390/diagnostics11050762 33922858 PMC8146750

[3] Evans CM Fingerlin TE Schwarz MI Lynch D Kurche J Warg L. Idiopathic pulmonary fibrosis: a genetic disease that involves mucociliary dysfunction of the peripheral airways. Physiol Rev. (2016) 96:1567–91. 10.1152/physrev.00004.2016 27630174 PMC5243224

[4] Chambers RC Mercer PF. Mechanisms of alveolar epithelial injury, repair, and fibrosis. Ann Am Thorac Soc. (2015) 12:S16–20. 10.1513/AnnalsATS.201410-448MG 25830828 PMC4430974

[5] Wolters PJ Collard HR Jones KD. Pathogenesis of idiopathic pulmonary fibrosis. Annu Rev Pathol. (2014) 9:157–79. 10.1146/annurev-pathol-012513-104706 24050627 PMC4116429

[6] Salton F Ruaro B Confalonieri P Confalonieri M. Epithelial-mesenchymal transition: a major pathogenic driver in idiopathic pulmonary fibrosis? Medicina. (2020) 56:608. 10.3390/medicina56110608 33202716 PMC7697350

[7] Kaur A Mathai SK Schwartz DA. Genetics in idiopathic pulmonary fibrosis pathogenesis, prognosis, and treatment. Front Med. (2017) 4:154. 10.3389/fmed.2017.00154 28993806 PMC5622313

[8] Yonemaru M Kasuga I Kusumoto H Kunisawa A Kiyokawa H Kuwabara S. Elevation of antibodies to cytomegalovirus and other herpes viruses in pulmonary fibrosis. Eur Respir J. (1997) 10:2040–5. 10.1183/09031936.97.10092040 9311499

[9] Vergnon JM Vincent M de The G Mornex JF Weynants P Brune J. Cryptogenic fibrosing alveolitis and Epstein-Barr virus: an association? Lancet. (1984) 2:768–71. 10.1016/S0140-6736(84)90702-5 6148520

[10] Bando M Ohno S Oshikawa K Takahashi M Okamoto H Sugiyama Y. Infection of TT virus in patients with idiopathic pulmonary fibrosis. Respir Med. (2001) 95:935–42. 10.1053/rmed.2001.1151 11778789

[11] Ueda T Ohta K Suzuki N Yamaguchi M Hirai K Horiuchi T. Idiopathic pulmonary fibrosis and high prevalence of serum antibodies to hepatitis C virus. Am Rev Respir Dis. (1992) 146:266–8. 10.1164/ajrccm/146.1.266 1320820

[12] Moore BB Moore TA. Viruses in idiopathic pulmonary fibrosis. Etiol Exacerbat Ann Am Thorac Soc. (2015) 12:S186–92. 10.1513/AnnalsATS.201502-088AW 26595738 PMC4722834

[13] Wootton SC Kim DS Kondoh Y Chen E Lee JS Song JW. Viral infection in acute exacerbation of idiopathic pulmonary fibrosis. Am J Respir Crit Care Med. (2011) 183:1698–702. 10.1164/rccm.201010-1752OC 21471095 PMC3136996

[14] Raghu G Anstrom KJ King TE Jr. Lasky JA Martinez FJ. Prednisone, azathioprine, and N-acetylcysteine for pulmonary fibrosis. New Engl J Med. (2012) 366:1968–77. 10.1056/NEJMoa1113354 22607134 PMC3422642

[15] Janda JM Abbott SL. 16S rRNA gene sequencing for bacterial identification in the diagnostic laboratory: pluses, perils, and pitfalls. J Clin Microbiol. (2007) 45:2761–4. 10.1128/JCM.01228-07 17626177 PMC2045242

[16] Molyneaux PL Cox MJ Willis-Owen SA Mallia P Russell KE Russell AM. The role of bacteria in the pathogenesis and progression of idiopathic pulmonary fibrosis. Am J Respir Crit Care Med. (2014) 190:906–13. 10.1164/rccm.201403-0541OC 25184687 PMC4299577

[17] Garzoni C Brugger SD Qi W Wasmer S Cusini A Dumont P. Microbial communities in the respiratory tract of patients with interstitial lung disease. Thorax. (2013) 68:1150–6. 10.1136/thoraxjnl-2012-202917 23945167 PMC3841796

[18] Friaza V la Horra C Rodriguez-Dominguez MJ Martin-Juan J Canton R Calderon EJ. Metagenomic analysis of bronchoalveolar lavage samples from patients with idiopathic interstitial pneumonia and its antagonic relation with Pneumocystis jirovecii colonization. J Microbiol Methods. (2010) 82:98–101. 10.1016/j.mimet.2010.03.026 20382190

[19] Takahashi Y Saito A Chiba H Kuronuma K Ikeda K Kobayashi T. Impaired diversity of the lung microbiome predicts progression of idiopathic pulmonary fibrosis. Respir Res. (2018) 19:34. 10.1186/s12931-018-0736-9 29486761 PMC6389110

[20] Molyneaux PL Cox MJ Wells AU Kim HC Ji W Cookson WO. Changes in the respiratory microbiome during acute exacerbations of idiopathic pulmonary fibrosis. Respir Res. (2017) 18:29. 10.1186/s12931-017-0511-3 28143484 PMC5286769

[21] Han MK Zhou Y Murray S Tayob N Noth I Lama VN. Lung microbiome and disease progression in idiopathic pulmonary fibrosis: an analysis of the COMET study. Lancet Respir Med. (2014) 2:548–56. 10.1016/S2213-2600(14)70069-4 24767767 PMC4142525

[22] Kitsios GD Rojas M Kass DJ Fitch A Sembrat JC Qin S. Microbiome in lung explants of idiopathic pulmonary fibrosis: a case-control study in patients with end-stage fibrosis. Thorax. (2018) 73:481–4. 10.1136/thoraxjnl-2017-210537 28802277 PMC6850567

[23] Wanger J Clausen JL Coates A Pedersen OF Brusasco V Burgos F. Standardisation of the measurement of lung volumes. Eur Respir J. (2005) 26:511–22. 10.1183/09031936.05.00035005 16135736

[24] Miller MR Hankinson J Brusasco V Burgos F Casaburi R Coates A. Standardisation of spirometry. Eur Respir J. (2005) 26:319–38. 10.1183/09031936.05.00034805 16055882

[25] Macintyre N Crapo RO Viegi G Johnson DC van der Grinten CP Brusasco V. Standardisation of the single-breath determination of carbon monoxide uptake in the lung. Eur Respir J. (2005) 26:720–35. 10.1183/09031936.05.00034905 16204605

[26] Holland AE Spruit MA Troosters T Puhan MA Pepin V Saey D. An official European Respiratory Society/American Thoracic Society technical standard: field walking tests in chronic respiratory disease. Eur Respir J. (2014) 44:1428–46. 10.1183/09031936.00150314 25359355

[27] Klindworth A Pruesse E Schweer T Peplies J Quast C Horn M. Evaluation of general 16S ribosomal RNA gene PCR primers for classical and next-generation sequencing-based diversity studies. Nucleic Acids Res. (2013) 41:e1. 10.1093/nar/gks808 22933715 PMC3592464

[28] Kozich JJ Westcott SL Baxter NT Highlander SK Schloss PD. Development of a dual-index sequencing strategy and curation pipeline for analyzing amplicon sequence data on the MiSeq Illumina sequencing platform. Appl Environ Microbiol. (2013) 79:5112–20. 10.1128/AEM.01043-13 23793624 PMC3753973

[29] Fadrosh DW Ma B Gajer P Sengamalay N Ott S Brotman RM. An improved dual-indexing approach for multiplexed 16S rRNA gene sequencing on the Illumina MiSeq platform. Microbiome. (2014) 2:6. 10.1186/2049-2618-2-6 24558975 PMC3940169

[30] Magoc T Salzberg SL FLASH. fast length adjustment of short reads to improve genome assemblies. Bioinformatics. (2011) 27:2957–63. 10.1093/bioinformatics/btr507 21903629 PMC3198573

[31] Li W Fu L Niu B Wu S Wooley J. Ultrafast clustering algorithms for metagenomic sequence analysis. Brief Bioinform. (2012) 13:656–68. 10.1093/bib/bbs035 22772836 PMC3504929

[32] Schloss PD Westcott SL Ryabin T Hall JR Hartmann M Hollister EB. Introducing mothur: open-source, platform-independent, community-supported software for describing and comparing microbial communities. Appl Environ Microbiol. (2009) 75:7537–41. 10.1128/AEM.01541-09 19801464 PMC2786419

[33] Caporaso JG Kuczynski J Stombaugh J Bittinger K Bushman FD Costello EK. QIIME allows analysis of high-throughput community sequencing data. Nat Methods. (2010) 7:335. 10.1038/nmeth.f.303 20383131 PMC3156573

[34] Gotelli NJ. Colwell RKJBdfim, assessment. Estimat Species Richness. (2011) 12:35. 10.1080/14452294.2011.11649538

[35] Magurran AE. Measuring Biological Diversity. John Wiley & Sons (2013). p. 108–9.

[36] Lande R. Statistics and partitioning of species diversity, and similarity among multiple communities. Oikos. (1996) 1996:5–13. 10.2307/3545743

[37] Lozupone C Lladser ME Knights D Stombaugh J Knight R. UniFrac: an effective distance metric for microbial community comparison. ISME J. (2011) 5:169–72. 10.1038/ismej.2010.133 20827291 PMC3105689

[38] Aroutcheva A Gariti D Simon M Shott S Faro J Simoes JA. Defense factors of vaginal lactobacilli. Am J Obstet Gynecol. (2001) 185:375–9. 10.1067/mob.2001.115867 11518895

[39] Walter J. Ecological role of lactobacilli in the gastrointestinal tract: implications for fundamental and biomedical research. Appl Environ Microbiol. (2008) 74:4985–96. 10.1128/AEM.00753-08 18539818 PMC2519286

[40] Raghu G Freudenberger TD Yang S Curtis JR Spada C Hayes J. High prevalence of abnormal acid gastro-oesophageal reflux in idiopathic pulmonary fibrosis. Eur Respir J. (2006) 27:136–42. 10.1183/09031936.06.00037005 16387946

[41] Harata G He F Hiruta N Kawase M Kubota A Hiramatsu M. Intranasal administration of Lactobacillus rhamnosus GG protects mice from H1N1 influenza virus infection by regulating respiratory immune responses. Lett Appl Microbiol. (2010) 50:597–602. 10.1111/j.1472-765X.2010.02844.x 20438620

[42] Agostini C Gurrieri C. Chemokine/cytokine cocktail in idiopathic pulmonary fibrosis. Proc Am Thorac Soc. (2006) 3:357–63. 10.1513/pats.200601-010TK 16738201

[43] Van der Meulen R Adriany T Verbrugghe K De Vuyst LJAEM. Kinetic analysis of bifidobacterial metabolism reveals a minor role for succinic acid in the regeneration of NAD+ through its growth-associated production. Appl Environ Microbiol. (2006) 72:5204–10. 10.1128/AEM.00146-06 16885266 PMC1538715

[44] Smith SM Eng RH Buccini F. Use of D-lactic acid measurements in the diagnosis of bacterial infections. J Infect Dis. (1986) 154:658–64. 10.1093/infdis/154.4.658 3528318

[45] Kottmann RM Kulkarni AA Smolnycki KA Lyda E Dahanayake T Salibi R. Lactic acid is elevated in idiopathic pulmonary fibrosis and induces myofibroblast differentiation via pH-dependent activation of transforming growth factor-beta. Am J Respir Crit Care Med. (2012) 186:740–51. 10.1164/rccm.201201-0084OC 22923663 PMC3480515

[46] Knippenberg S Ueberberg B Maus R Bohling J Ding N Tort Tarres M. Streptococcus pneumoniae triggers progression of pulmonary fibrosis through pneumolysin. Thorax. (2015) 70:636–46. 10.1136/thoraxjnl-2014-206420 25964315 PMC6729139

[47] Maus UA Srivastava M Paton JC Mack M Everhart MB Blackwell TS. Pneumolysin-induced lung injury is independent of leukocyte trafficking into the alveolar space. J Immunol. (2004) 173:1307–12. 10.4049/jimmunol.173.2.1307 15240724

[48] Cho SJ Moon JS Nikahira K Yun HS Harris R Hong KS. GLUT1-dependent glycolysis regulates exacerbation of fibrosis via AIM2 inflammasome activation. Thorax. (2020) 75:227–36. 10.1136/thoraxjnl-2019-213571 31822523 PMC7063401

[49] Sze MA Dimitriu PA Hayashi S Elliott WM McDonough JE Gosselink JV. The lung tissue microbiome in chronic obstructive pulmonary disease. Am J Respir Crit Care Med. (2012) 185:1073–80. 10.1164/rccm.201111-2075OC 22427533 PMC3359894

[50] Greathouse KL White JR Vargas AJ Bliskovsky VV Beck JA von Muhlinen N. Interaction between the microbiome and TP53 in human lung cancer. Genome Biol. (2018) 19:123. 10.1186/s13059-018-1501-6 30143034 PMC6109311

