# Supplementary material for: Lung Tissue Microbiome Is Associated With Clinical Outcomes of Idiopathic Pulmonary Fibrosis
Source: Front Med (Lausanne). 2021 Oct 18;8:744523. doi: 10.3389/fmed.2021.744523 (PMC8559550; doi:10.3389/fmed.2021.744523)
Supplement: Supplementary file 1 [file Data_Sheet_1.docx]

Supplementary Material

**Lung tissue microbiome is associated with clinical outcomes of idiopathic pulmonary fibrosis**

Hee-Young Yoon, Su-Jin Moon, Jin Woo Song

Department of Pulmonary and Critical Care Medicine, Asan Medical Center, University of Ulsan College of Medicine, Seoul, Republic of Korea

**Figure legends**

**Figure A1.** Comparison of alpha diversity indexes between patients with IPF and controls


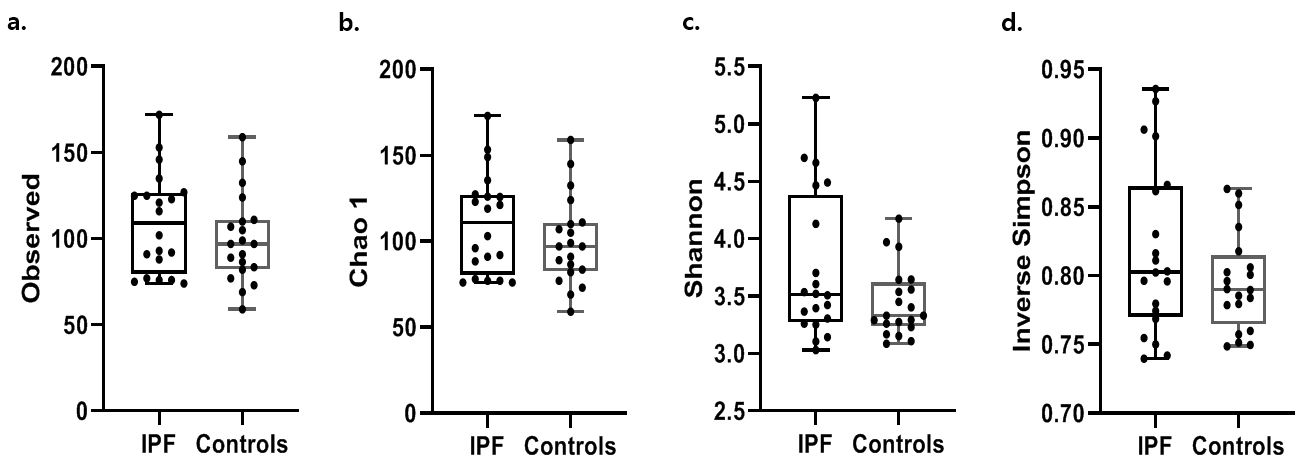


(a) Observed operational taxonomic units, (b) Chao 1, (c) Shannon index, and (d) Inverse Simpson index. Box plot represents minimum, first quartile, median, third quartile, and maximum of alpha indexes.

**Figure A2.** Comparison of microbial composition between patients with IPF and controls.


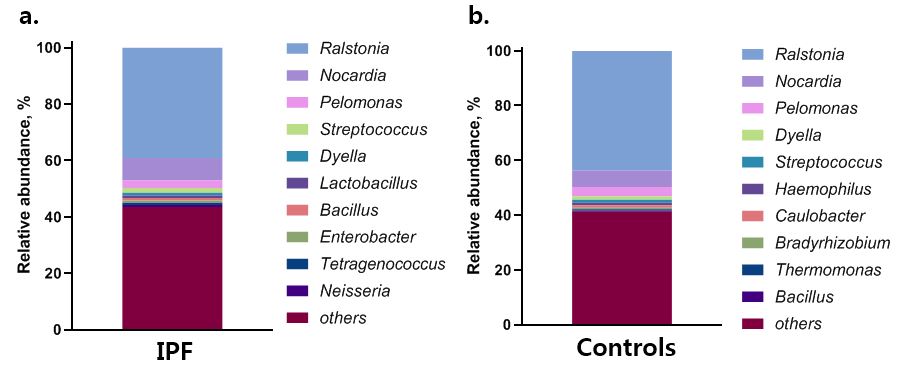


(a) IPF, (b) control. IPF, idiopathic pulmonary fibrosis

**Figure A3.** Comparison of alpha diversity indexes between non-survivors and survivors of IPF.


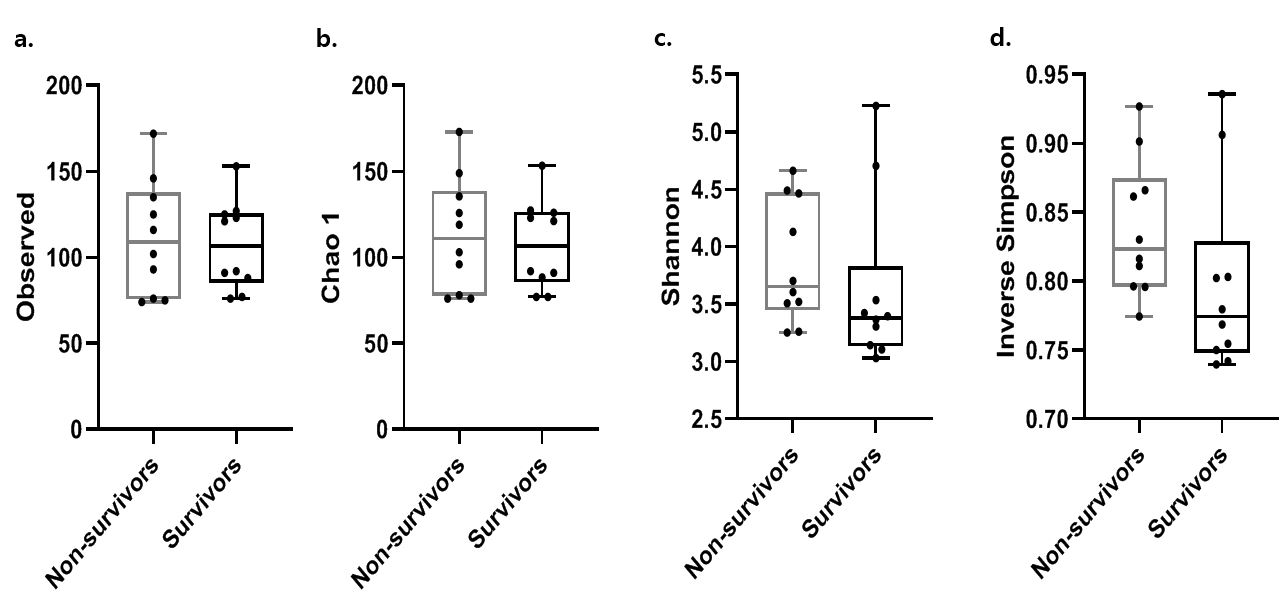


(a) Observed operational taxonomic units, (b) Chao 1, (c) Shannon index, and (d) Inverse Simpson index. Box plot shows minimum, first quartile, median, third quartile, and maximum alpha indexes.

**Figure A4.** Comparison of microbial composition between non-survivors and survivors of IPF.


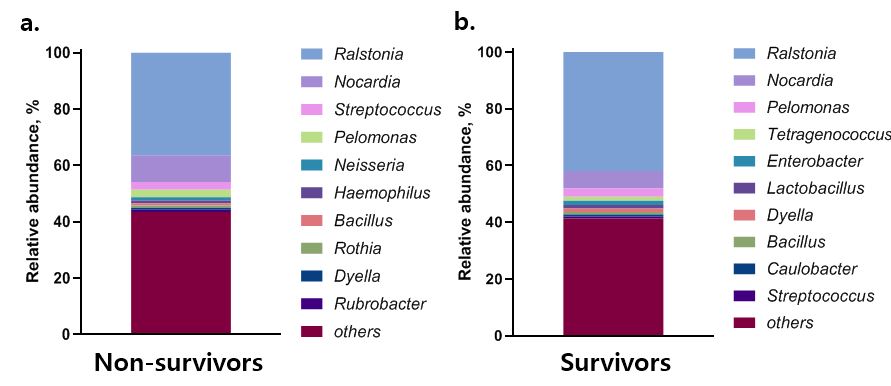


(a) Non-survivors. (b) Survivors.

**Table A1.** Correlation between microbial relative abundance and lung function in patients with IPF

|  |  | FVC | DLco | TLC |
| --- | --- | --- | --- | --- |
| *Curvibacter* | *r-value* | 0.590 | 0.181 | 0.240 |
|  | *p-value* | 0.002 | 0.444 | 0.307 |
| *Thermomonas* | *r-value* | 0.373 | 0.452 | 0.163 |
|  | *p-value* | 0.105 | 0.046 | 0.493 |
| *Thioprofundum* | *r-value* | 0.458 | 0.031 | 0.181 |
|  | *p-value* | 0.042 | 0.898 | 0.444 |
| *Granulicatella* | *r-value* | 0.133 | 0.075 | 0.542 |
|  | *p-value* | 0.545 | 0.753 | 0.014 |
| *Rhodoferax* | *r-value* | 0.222 | 0.022 | 0.461 |
|  | *p-value* | 0.346 | 0.927 | 0.041 |
| *Anoxybacillus* | *r-value* | -0.509 | -0.238 | 0.020 |
|  | *p-value* | 0.022 | 0.312 | 0.932 |
| *Enterococcus* | *r-value* | -0.593 | -0.185 | 0.045 |
|  | *p-value* | 0.006 | 0.434 | 0.851 |
| *Akkermansia* | *r-value* | -0.505 | -0.250 | -0.400 |
|  | *p-value* | 0.023 | 0.278 | 0.080 |
| *Clostridium* | *r-value* | -0.445 | -0.215 | -0.115 |
|  | *p-value* | 0.049 | 0.362 | 0.628 |
| *Peptoniphilus* | *r-value* | 0.245 | 0.504 | 0.119 |
|  | *p-value* | 0.681 | 0.258 | 0.239 |

DLco, diffusing capacity of the lung for carbon monoxide; FVC, forced vital capacity; IPF, idiopathic pulmonary fibrosis; TLC, total lung capacity.

**Table A2.** Correlation between microbial relative abundance and exercise capacity in patients with IPF

|  |  | 6MWD | Resting SpO_2_ | Lowest SpO_2_ |
| --- | --- | --- | --- | --- |
| *Granulicatella* | *r-value* | -0.254 | 0.284 | 0.489 |
|  | *p-value* | 0.279 | 0.224 | 0.029 |
| *Rhodoferax* | *r-value* | -0.044 | 0.584 | 0.461 |
|  | *p-value* | 0.855 | 0.007 | 0.041 |
| *Lactobacillus* | *r-value* | -0.022 | 0.258 | 0.488 |
|  | *p-value* | 0.927 | 0.271 | 0.029 |
| *Aquabacterium* | *r-value* | 0.616 | 0.352 | 0.379 |
|  | *p-value* | 0.004 | 0.128 | 0.099 |
| *Nakamurella* | *r-value* | 0.579 | 0.51 | 0.052 |
|  | *p-value* | 0.007 | 0.022 | 0.829 |
| *Acidocella* | *r-value* | 0.135 | 0.512 | 0.110 |
|  | *p-value* | 0.569 | 0.021 | 0.4646 |
| *Microbulbifer* | *r-value* | 0.303 | 0.499 | 0.335 |
|  | *p-value* | 0.194 | 0.025 | 0.148 |
| *Peptoniphilus* | *r-value* | 0.606 | 0.506 | 0.192 |
|  | *p-value* | 0.005 | 0.023 | 0.418 |
| *Lactococcus* | *r-value* | 0.018 | 0.597 | 0.67 |
|  | *p-value* | 0.941 | 0.005 | 0.001 |
| *Fusobacterium* | *r-value* | -0.464 | -0.007 | 0.121 |
|  | *p-value* | 0.039 | 0.975 | 0.611 |
| *Anaerococcus* | *r-value* | -0.481 | 0.053 | 0.005 |
|  | *p-value* | 0.032 | 0.823 | 0.984 |
| *Phycicoccus* | *r-value* | -0.495 | -0.2 | -0.057 |
|  | *p-value* | 0.027 | 0.397 | 0.812 |
| *Pedobacter* | *r-value* | -0.058 | -0.276 | -0.456 |
|  | *p-value* | 0.808 | 0.239 | 0.043 |

6MWD, 6-minute walk test distance; IPF, idiopathic pulmonary fibrosis; SpO_2_, oxygen saturation.

**Table A3.** Correlation between microbial relative abundance and changes in lung function in patients with IPF

|  |  | Δ FVC | Δ DLco | Δ TLC |
| --- | --- | --- | --- | --- |
| *Streptococcus* | *r-value* | -0.130 | -0.783 | -0.117 |
|  | *p-value* | 0.439 | <0.001 | 0.645 |
| *Lactobacillus* | *r-value* | 0.219 | -0.045 | 0.652 |
|  | *p-value* | 0.353 | 0.858 | 0.003 |
| *Staphylococcus* | *r-value* | 0.067 | 0.129 | 0.499 |
|  | *p-value* | 0.779 | 0.610 | 0.035 |
| *Bifidobacterium* | *r-value* | -0.041 | 0.605 | 0.165 |
|  | *p-value* | 0.863 | 0.008 | 0.513 |
| *Granulicatella* | *r-value* | 0.556 | 0.417 | 0.473 |
|  | *p-value* | 0.011 | 85.000 | 0.047 |
| *Selenomonas* | *r-value* | 0.407 | 0.157 | 0.542 |
|  | *p-value* | 0.075 | 0.534 | 0.020 |
| *Paracoccus* | *r-value* | 0.506 | 0.543 | 0.375 |
|  | *p-value* | 0.023 | 0.020 | 0.125 |
| *Novosphingobium* | *r-value* | -0.463 | -0.315 | -0.124 |
|  | *p-value* | 0.040 | 0.203 | 0.624 |

DLco, diffusing capacity of the lung for carbon monoxide; FVC, forced vital capacity; IPF, idiopathic pulmonary fibrosis; TLC, total lung capacity; Δ, decline rate for one year

**Table A4.** Correlation between microbial relative abundance and changes in exercise capacity in patients with IPF

|  |  | Δ 6MWD | Δ Resting SpO_2_ | Δ Lowest SpO_2_ |
| --- | --- | --- | --- | --- |
| *Enterobacter* | *r-value* | -0.103 | -0.516 | -0.085 |
|  | *p-value* | 0.684 | 0.028 | 0.738 |
| *Staphylococcus* | *r-value* | 0.486 | 0.116 | 0.538 |
|  | *p-value* | 0.041 | 0.648 | 0.021 |
| *Variovorax* | *r-value* | 0.525 | 0.323 | 0.321 |
|  | *p-value* | 0.025 | 0.191 | 0.194 |
| *Beijerinckia* | *r-value* | -0.218 | 0.472 | -0.183 |
|  | *p-value* | 0.385 | 0.048 | 0.468 |
| *Legionella* | *r-value* | -0.594 | -0.436 | -0.17 |
|  | *p-value* | 0.009 | 0.071 | 0.499 |
| *Anoxybacillus* | *r-value* | -0.666 | -0.037 | -0.324 |
|  | *p-value* | 0.003 | 0.885 | 0.19 |
| *Acidocella* | *r-value* | -0.589 | -0.144 | -0.151 |
|  | *p-value* | 0.01 | 0.57 | 0.549 |
| *Mycobacterium* | *r-value* | 0.454 | 0.537 | 0.441 |
|  | *p-value* | 0.058 | 0.022 | 0.067 |
| *Hyphomicrobium* | *r-value* | -0.615 | -0.307 | 0.074 |
|  | *p-value* | 0.007 | 0.215 | 0.769 |
| *Microbulbifer* | *r-value* | 0.067 | 0.477 | 0.308 |
|  | *p-value* | 0.791 | 0.045 | 0.214 |

6MWD, 6-minute walk test distance; IPF, idiopathic pulmonary fibrosis; SpO_2_, oxygen saturation; Δ, decline rate for one year
